# Autophagy: New Questions from Recent Answers

**DOI:** 10.5402/2012/738718

**Published:** 2012-12-30

**Authors:** Fulvio Reggiori

**Affiliations:** Department of Cell Biology and Institute of Biomembranes, University Medical Centre Utrecht, Heidelberglaan 100, Utrecht, The Netherlands

## Abstract

Macroautophagy (hereafter autophagy) is currently one of the areas of medical life sciences attracting a great interest because of its pathological implications and therapy potentials. The discovery of the *autophagy-related genes (ATGs)* has been the key event in this research field because their study has led to the acquisition of new knowledge about the mechanism of this transport pathway. In addition, the investigation of these genes in numerous model systems has revealed the central role that autophagy plays in maintaining the cell homeostasis. This process carries out numerous physiological functions, some of which were unpredicted and thus surprising. Here, we will review some of the questions about the mechanism and function of autophagy that still remain unanswered, and new ones that have emerged from the recent discoveries.

## 1. Introduction

**Figure 1:**
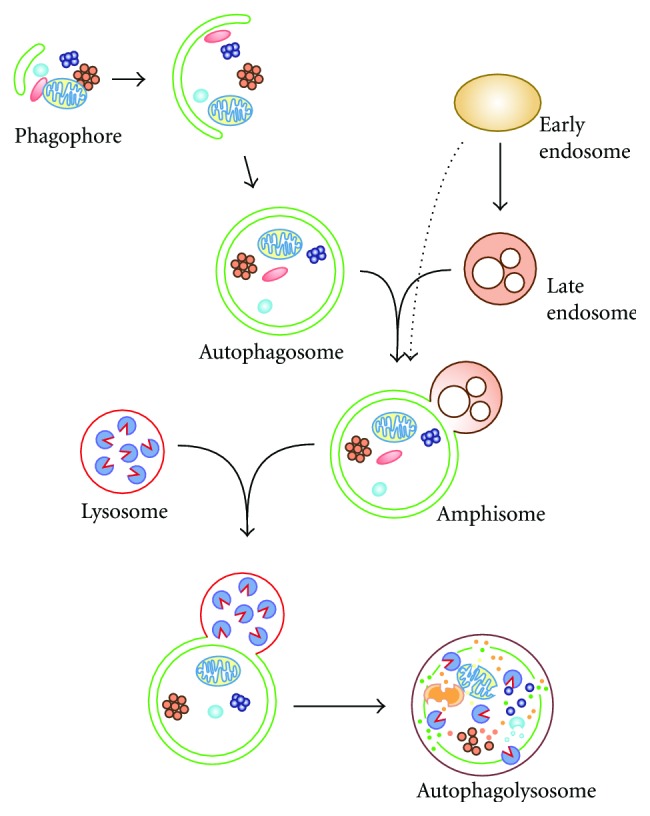
Schematic representation of the process of autophagy. Phagophores are the initial precursor structure of this transport pathway. These membrane cisterns are formed at the PAS by the Atg machinery, which also catalyzes their expansion into autophagosomes through the acquisition of extra lipid bilayers. During this latter event, the growing phagophore sequesters cytoplasmic components or specific structures depending on the autophagy-inducing conditions. The closure of the expanding phagophore leads to the formation of a double-membrane vesicle called an autophagosome, which contains the cargo targeted for degradation. The Atg machinery is then released from the surface and the complete autophagosomes, which initially fuse with endosomal compartments generating amphisomes. While the cargo material starts to be already turned over in the amphisomes, the exposure to hydrolases by fusion with lysosomes to form autolysosomes allows its complete degradation into basic metabolites such as amino acids and sugars, which are transported in the cytoplasm and used as an energy source or building blocks for the synthesis of new macromolecules. Adapted from [8, 9].

The basic mechanism of autophagy is the sequestration of the structure that has to be degraded by large cytoplasmic double-membrane vesicles called autophagosomes. The current model is that autophagosomes are formed by expansion and sealing of a small cistern known as the phagophore or isolation membrane (Figure 1) [1–5]. Once complete, they fuse with the mammalian lysosomes or plant and yeast vacuoles to expose their cargo to the hydrolytic interior of these compartments for degradation. In mammalian cells, this event is preceded by the fusion with vesicles of the endocytic pathway and/or endosomes, to form amphisomes (Figure 1) [6]. The metabolites generated in the lysosomes/vacuoles are subsequently transported in the cytoplasm and used as either an energy source or building blocks for the synthesis of new macromolecules. The phagophore is generated at a specialized site known as the phagophore assembly site or preautophagosomal structure (PAS) [1–5]. At this location, the key actors of this pathway, the *autophagy-related genes (ATGs)*, mediate the formation of the phagophore and its expansion into an autophagosome. Sixteen Atg proteins compose the conserved core Atg machinery that catalyses the formation of autophagosomes in all eukaryotes. The rest of the Atg proteins are organism-specific and most of them are involved in either the regulation of autophagy or dictating the specificity during selective types of autophagy. Autophagy has been considered for long time a nonselective process for bulk degradation of either long-lived proteins or cytoplasmic components during nutrient deprivation. Recent evidences, however, have revealed the existence of numerous types of selective autophagy used by the cell to specifically eliminate unwanted structures including organelles and invading microorganisms [7]. Under specific conditions, autophagosomes can thus exclusively sequester and degrade mitochondria (i.e., mitophagy), peroxisomes (i.e., pexophagy), endoplasmic reticulum (ER) (i.e., ER-phagy or reticulophagy), endosomes/lysosomes, lipid droplets (i.e., lipophagy), secretory granules (i.e., zymophagy), cytoplasmic aggregates and complexes (i.e., aggrephagy), ribosomes (i.e., ribophagy), invading pathogens (i.e., xenophagy) and so forth.

Because of its ability to rapidly eliminate unwanted structures, autophagy participates in a multitude of physiological processes essential to maintain cellular and organismal homeostasis such as the adaptation to starvation, cell differentiation and development, degradation of aberrant structures, turnover of superfluous or damaged organelles, tumorsuppression, innate and adaptive immunity, lifespan extension, and type II programmed cell death [10–13]. As a result, a defect or an impairment in this pathway leads to severe illnesses including neurodegenerative, cardiovascular, chronic inflammatory, muscular and autoimmune diseases, and some malignancies. Crucially, it has also been shown that autophagy could be a potential therapy to prevent or cure particular diseases, including specific types of tumors, muscular dystrophies, neurodegenerative disorders, and selected infections [14–19].

## 2. The Atg Proteins and the Autophagosome Biogenesis

### 2.1. The Autophagosome Formation

**Figure 2:**
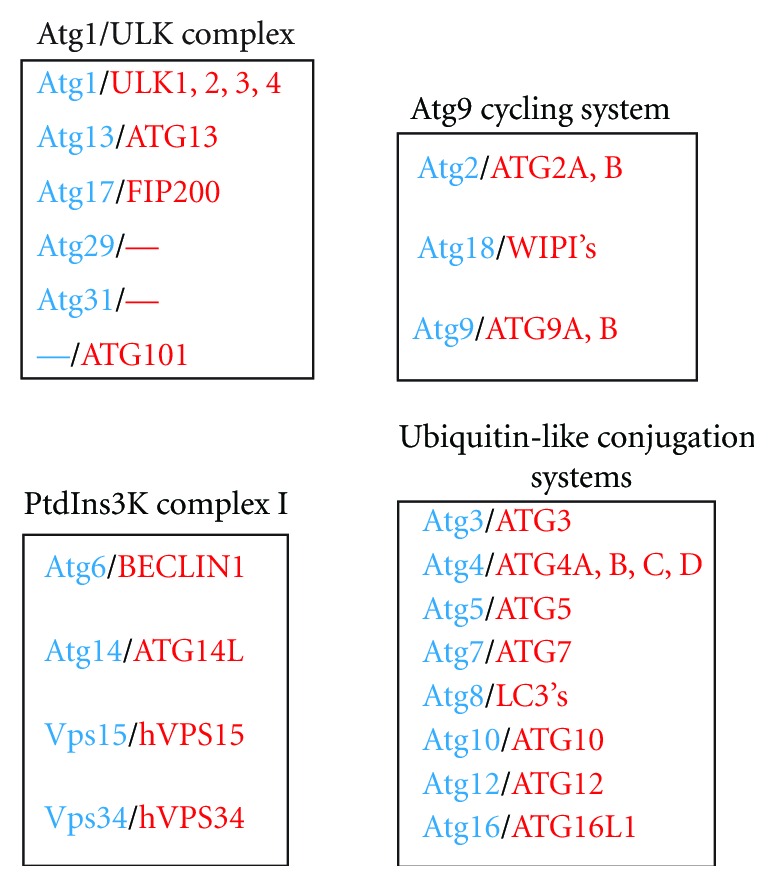
The organisation of the Atg machinery in functional groups. Yeast *ATGs* are in blue while the mammalians counterparts, which in few cases comprise few paralogues, are in red. The WIPI's is a protein family which comprises 4 members: WIPI1, WIPI2, WIPI3, and WIPI4 [20]. Three of them have been shown to be involved in autophagy [20–22]. The LC3's is a protein family that comprise 6 proteins: LC3A, LC3B, LC3C, GABARAPL1, GABARAPL2, and GABARAPL3 [23]. All of them associate with autophagosomes [23, 24]. The yeast Atg1 complex contains two subunits, Atg29 and Atg31, which do not have homologues in high eukaryotes. In contrast, the mammalian complex possesses a component, Atg101, which is not found in yeast.

A central objective in the field of autophagy is to assign a function to the Atg proteins, that is, how these factors assemble, rearrange, and expand membranes into an autophagosome. Although the exact molecular role of the core Atg proteins remains unknown, they have been classified into five Atg functional groups principally based on interactions: the Atg1/Ulk kinase complex, the Atg9 cycling system, the autophagy-specific phosphatidylinositol 3-kinase (PtdIns3K) complex I, and the two ubiquitin-like conjugation systems (Figure 2).

#### 2.1.1. The Atg1/ULK Complex

Atg1 is a serine/threonine protein kinase that has a key role in autophagy induction [25]. Different proteins associate to form a complex with Atg1. In yeast, this kinase is associated with Atg13, Atg17 and two nonconserved subunits, Atg29 and Atg31, while ULK1 and ULK2, two mammalian redundant Atg1 homologues, associate with mATG13 and FIP200, the counterparts of Atg13 and Atg17, respectively, and the nonconserved component ATG101 (Figure 2) [26–31]. The signaling cascade centered on the serine/threonine kinase mammalian target of rapamycin (mTOR) promotes cell growth and anabolism in presence of nutrients [32]. This pathway inhibits autophagy through direct modulation of the Atg1/ULK complex. In nutrient rich conditions, mTOR is associated with the Atg1/ULK complex via ULK1 or ULK2 and it maintains mATG13 phosphorylated [31, 33–36]. Under nutrient deprivation, mTOR dissociates from this complex provoking a dephosphorylation of ULK1 and ULK2 necessary for the activation of their kinase activity and subsequent phosphorylation of FIP200, mATG13, and ULK1/2 itself [35]. All these modifications are necessary to initiate autophagy.

#### 2.1.2. The PtdIns3K Complex I

This complex is formed by Vps34/hVPS34, Vps15/p115, Atg6/BECLIN1, and Atg14/ATG14L (Figure 2), and it is essential for the generation of PtdIns3P on autophagosomal membranes and for the progression of autophagy [37–39]. The role in autophagy of this lipid, which is found on the surface and interior of autophagosomes [40, 41], remains unclear. Nevertheless, one function is to recruit factors such as Atg18 to the PAS and possibly also to the phagophore. The formation of PtdIns3P depends on the activity of PtdIns3 kinase class III hVPS34, which is present on the surface of various organelles [42]. Atg14 is a subunit of the autophagy-specific PtdIns3K complex both in yeast and in mammals. There are at least two different Vps34-containing complexes in yeast [43], which, in addition to Vps34, Vps15, and Atg6/Vps30, also possess specific subunits: Atg14 and Vps38. These two last components direct the PtdIns3K complexes to specific locations where they generate the PtsIns3P pools essential for autophagy and endosomal trafficking, respectively. A similar situation also appears to be present in mammalian cells, with UVRAG being the homologue of Vps38 [38].

In mammalian cells, the PtdIns3K complex I also controls autophagy induction. When BECLIN1 self-associates or binds to BCL-XL/BCL-2, the lipid kinase activity of hVPS34 is inhibited as BECLIN1 is not part of the complex [37, 44, 45]. Upon nutrient deprivation, the JNK1 signaling pathway phosphorylates BCL-2 leading to its dissociation from BECLIN1, which permits this protein to interact with the PtdIns3K complex I stimulating PtdIns3P synthesis and autophagy induction [45, 46]. In parallel, autophagy positive regulators such as AMBRA1 and BIF-1 promote BECLIN1 association to hVPS34 [47–49].

#### 2.1.3. The Atg9 Cycling System

Atg9 is another protein that is found at an early stage of the PAS formation and it is the only integral membrane protein among the core Atg machinery [50]. It possesses six conserved transmembrane domains with the two cytoplasm-oriented termini, and it is essential for autophagy [51, 52]. Mammalian Atg9 (mATG9) localizes to the trans-Golgi network (TGN) in fed cells and partially to the late endosomes [52]. Upon autophagy induction by starvation, mATG9 relocates to the site where autophagosomes are generated, possibly the PAS and/or phagophores [52, 53]. It has recently been shown, however, that mATG9 positive membranes do interact dynamically with the autophagosomal intermediate rather than becoming integral part of them [53]. Similarly, yeast Atg9 is located at the PAS and in several cytoplasmic structures, which are likely to be directly derived from the Golgi [54–56]. The high mobile cytoplasmic structures are probably 30–60 nm vesicles, while the less mobile appear to be constituted by clusters of vesicles and tubules [56], which have been named Atg9 reservoirs [54] and have also been observed in mammalian cells [53]. As in mammalian cells, yeast Atg9 also cycles between the cytoplasmic pools and the PAS but it seems to arrive at the early stage of the formation of this structure and to be retrieved when an autophagosome is formed [54, 56].

Several factors regulate Atg9 trafficking including the Atg1/ULK and PtdIns3K complexes [52, 57]. Two other core Atg proteins, Atg2 and Atg18, are involved in Atg9 cycling. In particular, they appear to mediate Atg9 retrieval from the PAS [57, 58]. In yeast, Atg2 and Atg18 form a cytoplasmic complex [59]. While the formation of this complex does not require PtdIns3P, the presence of this lipid at the PAS is necessary for its recruitment to this site [59]. This is achieved through the capacity of Atg18 to directly bind PtsIns3P [59]. Mammals possess 4 Atg18 homologues: WD-repeat protein interacting with phosphoinositides 1 (WIPI1), WIPI2, WIPI3, and WIPI4 [20]. Three of them, WIPI1, WIPI2, and WIPI4 have been implicated in autophagy [20–22]. Recently, two mammalian Atg2 homologs, Atg2A and Atg2B, have been identified and both are required for autophagy [58]. Interestingly, human WIPI4 interacts with Atg2A and Atg2B as well as *Caenorhabditis elegans* EPG-6/WIPI4 with ATG2 [21, 60]. These observations suggest that WIPI4/EPG-6 and yeast Atg18 overlap in their role in autophagy by carrying out the functional interconnections with Atg2 [58].

#### 2.1.4. The Atg12 and Atg8/LC3 Conjugation Systems

The elongation of the phagophores and the completion/sealing of autophagosomes appear to rely on the function of these two ubiquitin-like systems (Figure 2). Atg12, an ubiquitin-like molecule, is covalently conjugated to Atg5 through the activity of Atg7 and Atg10, an E1- and an E2-like enzyme, respectively [23, 61–63]. The Atg12-Atg5 complex subsequently associates with Atg16 forming a large oligomer that localizes to both the PAS and the phagophore via Atg16 [64]. The function of the Atg12-Atg5·Atg16 oligomer in autophagy is unclear, but it seems that it acts as an E3 ligase for the generation of the lipidated form of Atg8/LC3 [65]. Atg8 is a second ubiquitin-like protein participating in autophagy. While yeast has only one copy of Atg8, mammalian cells have 6 homologues and all are involved in autophagy [23, 24, 62]. Atg8 is posttranslationally processed by the specific cysteine protease Atg4, which cleaves its C-terminal amino acids exposing a glycine residue. Through another ubiquitylation-like reaction mediated by Atg7 and the E2-like enzyme Atg3, Atg8 is covalently conjugated to phosphatidylethanolamine (PE). This lipidation promotes Atg8 recruitment and association with autophagosomal membranes [23, 61–63]. In contrast to the rest of the Atg proteins, which are mainly present on the surface of autophagosomes, Atg8 is found inside and outside these vesicles. When an autophagosome is completed, Atg4 cleaves the Atg8-PE pool on the surface releasing Atg8 back in the cytoplasm for reuse. Atg8 has been shown to be essential for autophagosome formation possibly by mediating tethering and fusion of membranes [66, 67]. These data, however, are controversial [68]. What is clear is that the Atg8 population associated with autophagosome inner membrane is essential for the selective sequestration of specific cargoes and together with them it is degraded in the lysosome/vacuole lumen (see above).

In addition to the Atg proteins, additional factors play a crucial role in the autophagosome biogenesis especially in high eukaryotes. Important ones include AMBRA1 [47, 48], DFCP1 [69, 70], and VMP1 [70, 71]. The detailed discussion of the role of these proteins as well as their functional relationship with the different Atg functional groups is not the subject of this review, and they have been extensively presented elsewhere [1–5].

Almost all the Atg proteins are cytosolic and associate to form the PAS by interacting with other Atg components and/or lipids upon autophagy induction [3, 50, 72]. Most of the studies about the PAS have been done in yeast and they have revealed that the core Atg proteins assemble following a hierarchical order and form this autophagosomal precursor [3, 50, 72]. Recent evidences have shown that the PAS and the principles of this ordered recruitment are conserved in mammals [70]. While these works have proposed a model where one Atg protein is at the top of the hierarchical recruitment cascade, studies on the selective elimination of either mitochondria or *Salmonella* indicate that the Atg proteins can be grouped into clusters, which independently assemble to form the PAS [73–75]. Interestingly, these clusters mirror almost entirely the organization in functional groups of the Atg proteins.

One of the enigmas in the field of autophagy is the origin of the lipid bilayers composing autophagosomes. Several cellular compartments, including the ER, Golgi, endosomes, and the plasma membrane, have been implicated as the possible source of the autophagosomal membranes by a series of recent studies [76–78]. This apparent discrepancy between the different reports could be due to the ability of cells to derive the membranes from the most suitable reservoirs depending on the tissues and conditions triggering autophagy. Thus in a tissue under a specific stress, autophagy would be supplied with membranes from an organelle that could guarantee the delivery of a large amounts of lipids [76–78]. An alternative option would be that the diverse Atg functional clusters that come together to generate the PAS (and the phagophore) are associated to membranes derived from different compartments explaining why endosomes, the plasma membrane, and the Golgi have all been shown to contribute to the formation of the early autophagosomal intermediates [76]. This model would also explain the involvement of proteins mediating membranes fusion such as the SNAREs in the early stages of autophagosome biogenesis [68, 79]. Thus in addition to unveiling the molecular function of each Atg protein, the challenge for the future will be to understand the mechanism underlying the integrated interaction between the different Atg functional groups, which will probably also be key in uncovering the events leading to the assembly of the autophagosomal membranes and possibly identify new mechanisms for the regulation of autophagy.

The major amount of lipids, however, is required for the expansion of the phagophore into an autophagosome. The current idea is that the ER plays a central role in this event because growing phagophores have been observed in close proximity of this organelle [69, 80, 81]. Contact sites between these two compartments have been detected [80, 81] and therefore it has been postulated that transfers could occur by direct lipid translocation from the ER to the nascent autophagosome. It remains to be proven whether this unidirectional passage of lipids between these two organelles indeed exists and how this is achieved.

### 2.2. The Autophagosome Completion

Autophagosomes are ready to fuse with the lysosome/vacuole once the vesicle membranes are sealed and the Atg machinery is disassembled and released back in the cytoplasm for reuse [72, 82]. Evidence for this latter event has been provided by the observation that Atg8/LC3, an ubiquitin-like protein that is covalently conjugated to autophagosomal membranes through a reversible linkage to phosphatidylethanolamine (PE), is not found on the surface of complete vesicles while it is abundantly detected on phagophores and nascent autophagosomes [83]. Accordingly, failure to release Atg8 form the autophagosome surface by Atg8-PE delipidation leads to an impairment of autophagy [84, 85]. Recently, it has been revealed that the turnover of phosphatidylinositol-3-phosphate (PtdIns3P), a lipid generated at the PAS and involved in the recruitment of Atg proteins to this location, is key in the disassembly of the Atg machinery from the surface of yeast autophagosomes [86]. This event is a requisite for the fusion of these carriers with the vacuole [86] indicating that the cell possesses a regulatory factor to avoid premature and potentially harmful fusion of incomplete double-membrane vesicles with the vacuole/lysosome. It remains to be identified this factor (or factors) that is able to sense the autophagosome completion and thus trigger PtdIns3P turnover, the Atg4-mediated processing of Atg8-PE, and the release of the rest of the Atg machinery.

## 3. Regulation of Autophagy

Autophagy can be induced by numerous environmental and cellular stresses. As a result several signaling molecules and cascades have been shown to be involved in the modulation of this pathway [4, 8, 87]. Biochemical and pharmacological experiments have highlighted the upstream effector role of Atg1/Ulk1 and PtdIns3K complexes in the transduction of these signals into the initiation of autophagosome biogenesis. Atg9 also appears to participate in the regulation of autophagy [88, 89].

The best-characterized regulator of autophagy is mTOR and as already introduced above it represses this pathway by principally blocking the activity of the Atg1/ULK1 complex through direct phosphorylation [4, 8, 87]. The activity of mTOR is stimulated by a variety of anabolic inputs that include the energy and nutrient status of the cell as well as the presence of amino acids and growth factors. Conversely, mTOR is inhibited when amino acids are scarce, growth factor signaling is reduced and/or ATP concentrations fall, and this results in a derepression of autophagosome biogenesis. The energy-sensing AMP-activated protein kinase (AMPK) and glucose-sensing protein kinase A (PKA) also regulate the Atg1/ULK1 complex by direct phosphorylation [90–92]. The molecular details of these regulations and the cross-talk between them remain to be elucidated.

Numerous molecules including interferon *γ* (IFN*γ*), tumor necrosis factor *α* (TNF*α*), and vitamin D, but also receptors such the toll-like receptors (TLRs) or the pattern recognition receptors (PPRs) have been shown to regulate autophagy as well [13, 93–95]. It is largely unknown how this is achieved but understanding these signaling mechanisms could have the added value of providing the knowledge essential for the development of either treatments or drugs for autophagy-based therapies to cure of specific diseases [14].

Some of the open questions regarding autophagy regulation have accurately been discussed in a recent compendium [96].

## 4. Cargo Recognition

In addition to the core Atg machinery, the selective types of autophagy rely on specific cargo-recognizing autophagy receptors that assure the cargo sequestration into autophagosomes. Autophagy receptors are defined as proteins being able to interact directly with both the structure that has to be specifically eliminated by autophagy and the pool of the Atg8/LC3 protein family members present in the internal surface of growing autophagosomes [7, 97]. This latter interaction is in most of the cases mediated through a specific sequence present in the autophagy receptors and commonly referred to as the LC3-interacting region (LIR) motif [98]. It has recently been shown that particular proteins possessing this motif including Atg1/Ulk1 are also directly turned over by autophagy without the necessity of having an autophagy receptor [30].

The autophagy receptors for the selective degradation of several complexes and organelles have been identified but others such as those for the specific turnover of the ER and ribosomes are still elusive [7, 97]. One emerging theme is that structures targeted for destruction are ubiquitinylated and a series of autophagy receptors such as p62/SQSTM1 and NBR1 with an ubiquitin-banding domain and a LIR motif, promote their sequestration into autophagosomes [7, 97, 98]. While these molecules preferentially recognize short ubiquitin chains [99], it is still unclear why they do not bind other cellular components carrying the same types of posttranslational modification. Central in understanding these specific elimination processes will be the identification of the E3 ligases and their eventual adaptors involved in marking the autophagy cargoes with ubiquitin. SMURF1 and STING appear to belong to these two classes of proteins [100, 101]. The investigation of proteins like these will provide information about how the cell senses and regulates the degradation of unwanted structures by autophagy.

Atg30 and Atg32 are two yeast autophagy receptors involved in pexophagy and mitophagy, respectively, which do not use the ubiquitin system to bind the targeted cargo but nevertheless their study has provided insights into possible mechanisms that could also be used by the E3 ligases [102–104]. These proteins are present on the surface of peroxisomes and mitochondria, respectively, and under mitophagy and peroxisome-inducing conditions they get phosphorylated by signaling cascades activated under these conditions [102, 105, 106]. The phosphorylation of Atg30 and Atg32 promotes the association and recruitment of Atg11, which in turn triggers the assembly of the Atg machinery mediating the formation of a double-membrane vesicle around the organelle [102–104]. Atg30 is present in *Pichia pastoris *but not in *Saccharomyces cerevisiae*, which uses a different molecule for perxophagy, that is, Atg36 [107]. Atg30 is a soluble protein that becomes phosphorylated when pexophagy is stimulated. This modification leads to its recruitment onto the peroxisome surface and its subsequent biding to Atg11 results in a selective engulfment of peroxisome by autophagosomes [107].

## 5. Against the Paradigms

### 5.1. More than a Degradative Pathway

For a long time autophagy has been considered a degradative transport route but recent discoveries have begun to change this view. The yeast cytosol-to-vacuole transport (Cvt) pathway is a biosynthetic selective type of autophagy that delivers a subset of hydrolases into the vacuole [108]. Shortly after synthesis, the proform of these hydrolases assembles into a large cytoplasmic oligomer, which is subsequently sequestered into a double-membrane vesicle that fuses with the vacuole. In the vacuole, the resident proteases cleave the profragment of the hydrolases composing the oligomer leading to both their activation and the disassembly of this structure [108].

For long the transport function of the Cvt pathway has been considered an exception in the field of autophagy. Recently it has been shown that the extracellular delivery of the cytosolic Acyl coenzyme-A-(CoA-) binding protein in the yeast *Pichia pastoris* and *Saccharomyces cerevisiae* (ACBP), and the social amoebae *Dictyostelium discoideum *(AcbA), which occurs under starvation conditions, is not mediated by the secretory pathway [109, 110]. The used unconventional transport route depends on the *ATG* and the Golgi ReAssembly and Stacking Protein (GRASP/Grh1) [109, 110], suggesting that autophagosomes could be the hallmark of this type of unconventional secretion. This notion is supported by work in yeast *S. cerevisiae* that has revealed that when this new transport route is triggered by starvation, Grh1 is recruited to membranous structures that are positive for Atg8 and Atg9 [111], and morphologically and molecularly resemble to precursor structures involved in autophagy [54]. Interestingly, the unconventional secretion of cytosolic IL-1*β* and HMGB1 by macrophages upon their stimulation with either starvation or lipopolysaccharides- (LPS-) treatment also requires the *ATG* and GRASP55, one of the paralogues of GRASP/Grh1, indicating that this process could be conserved among eukaryotes [112]. Additionally, autophagosomes expel engulfed material, mostly of plasma membrane origin, by fusing with the plasma membrane during the last stages of reticulocytes maturation into erythrocytes (intracellular turnover is not possible because lysosomes are absent in these cells) [113]. Finally, it has been hypothesized that picornaviruses exploit autophagosomes to secrete their newly synthesized virions [114] and while it was assumed that these viruses were somehow hijacking and diverting these carriers, one emerging possibility could be that they take advantage of an existing type of autophagy mediating the extracellular delivery of specific cytosolic components.

Under ER stress conditions that activate the unfolded protein response (UPR), yeast cells expand their ER volume to probably accommodate newly synthesized chaperones and to buffer the accumulation of unfolded proteins under UPR-inducing conditions. This phenomenon is accompanied by the formation and accumulation of autophagosomes that are densely and selectively packed with ER membranes [115]. Very surprisingly, the ER sequestration into autophagosomes and not its degradation is the crucial step allowing the cell to survive under these stress conditions [115]. While it remains totally unknown the fate of these autophagosomes, these data highlight the possibility that in specific situations autophagosomes could be persistent organelles rather than transport carriers, a notion somehow reminiscent with those infections where pathogens subvert autophagy to use autophagosomes as a platform for their intracellular replication [116].

### 5.2. The Unconventional Types of Autophagy

A completely new research area is the study of those forms of autophagy that do not require all the components of the core Atg machinery, which until recently were believed to be the absolute requirement for the generation of autophagosomes [117, 118]. One of the first reports describing one of these alternative processes of autophagy showed that when cells are subjected to particular stresses such as the treatment with the cytotoxic compound etoposide, they can form autophagosomes out of the Golgi and perform autophagy-mediated protein degradation in an ATG5-, ATG7-, ATG9-, and ATG16-independent way [119]. Nonetheless this pathway still requires ULK1/Atg1, FIP200/Atg17, BECLIN1/Atg6, and hVPS34/Vps34 [119]. BECLIN1, however, has been shown to be dispensable for autophagy in several situations, most of which involved treatment of cells with proapoptotic compounds such as the neurotoxin 1-methyl-4-phenylpyridinium, staurosporine, MK801, resveratrol, and Z18 [120–124]. The autophagy-specific PtdIns3K complex I controls autophagy induction and BECLIN1 can be part of it. When BECLIN1 self-associates or binds to Bcl-XL/Bcl-2, the lipid kinase activity of hVps34 is inhibited as BECLIN1 is not part of this complex [37, 44, 45]. Upon nutrient deprivation, the JNK1 signaling pathway phosphorylates Bcl-2 leading to its dissociation from BECLIN1, which permits this protein to interact with the PtdIns3K complex I stimulating PtdIns3P synthesis and autophagy induction [45, 46]. Because the BECLIN1-independent types of autophagy still entirely or partially require the generation of PtdIns3P [121, 125, 126], one possibility is that PtdIns3K complex I is stimulated in a different way under conditions that trigger this alternative pathway. A lot still need to be understood about the mechanism of these unconventional types of autophagy and future studies will also tell us why the cell utilizes them.

### 5.3. Nonautophagy-Related Functions of the Atg Proteins

One of the principal assumptions of the field of autophagy has been that the Atg proteins are involved in autophagosome biogenesis exclusively. Studies on the role of autophagy in immunity, especially in the context of specific viral and bacterial infections, have revealed a different picture. There are now several experimental findings showing that individual Atg proteins or Atg functional groups can also be part of other processes.

The ERAD tuning is a transport pathway out of the ER that mediates the rapid turnover of specific ERAD factors in the endosomal system [127–129]. In the ER SEL1L, a single transmembrane protein cargo receptor, binds and sorts these ERAD factors into vesicles called EDEMosomes [130]. The cytoplasmic tail of SEL1L binds to the nonlipidated form of LC3, that is, LC3-I, and as a result the EDEMosomes are LC3-I-positive [128, 130]. This notion of an unconventional use of LC3 by the ERAD tuning, which does not depend on an intact Atg machinery, has been reinforced by the observation that ATG5 and ATG7 are not involved in this pathway [128, 131] and the end product of the two autophagy conjugation systems to which these two proteins belong to, that is, lipidated Atg8/LC3 also known as LC3-II [62], is not present on the EDEMosomes [128]. The molecular function of LC3-I in the ERAD tuning is unclear and one hypothesis is that it acts as an adaptor to a not yet identified vesicle protein coat. Alternatively, the capacity of LC3 to associate with microtubules [132] could permit the EDEMosome to traffic following routes traced by the cytoskeleton. Coronavirus (CoV) cell infection is characterized by the formation of reticulovesicular networks of double-membrane vesicles (DMVs) and convoluted membranes, onto which replication-transcription complexes are associated. Studies with the mouse hepatitis virus (MHV), a CoV, have revealed that the *ATG5 *and *ATG7 *gene products are not required for the formation of the virus-induced DMVs and accordingly LC3-II is not present on these structures [131, 133]. In contrast LC3-I decorates the MHV-induced DMVs and the depletion of this protein blocks virus replication [131]. What has been shown is that CoV hijack the ERAD tuning by probably coopting SEL1L because this receptor also localizes to the MHV-induced DMVs and the virus replication is severely impaired when it is depleted [130]. Interestingly, the Equine Arteritis Virus (EAV), a member of the arterivirus virus family that belongs to the Nidoviralesorder like the CoV, is also hijacking the LC3-I-positive membranes of the ERAD tuning to replicate in host cells [134]. Finally and similarly to CoV, LC3-I (but not LC3-II) is associated to and essential for the formation of the intracellular inclusions of the *Chlamydia trachomatis* [135]. The generation of the *C. trachomatis *inclusions also does not require an intact Atg machinery [135, 136] but it remains unknown whether this bacterium is subverting the ERAD tuning as well.

*Brucella abortus* ensures its intracellular survival by forming the *Brucella*-containing vacuoles (BCVs), which traffic from the endocytic compartment to the ER where this bacterium proliferates [137]. The replication of *Brucella* in the ER is followed by conversion of the BCVs into compartments with autophagic features that have been named autophagic BCVs (aBCVs) [138]. The aBCVs formation is essential for both the intracellular life cycle and cell-to-cell spreading of *Brucella*, and requires proteins involved in the induction of autophagosome biogenesis such as ULK1, BECLIN1 and ATG14L, and the PtdIns3K activity [138]. The generation of the aBCVs, however, does not require proteins of the two conjugation systems like ATG5, ATG16L1, ATG4B, ATG7, and LC3B [138]. It remains to be understood whether this microbe is either subverting part of the Atg machinery or exploiting a pathway that uses a subset of the Atg proteins.

A somehow opposite situation is observed in osteoclasts. These cells resorb bone tissue by removing its mineralized matrix and breaking up the organic bone principally composed by collagen [139]. For this resorption, the osteoclasts form specialized plasma membrane protrusions, the ruffled borders, which are opposed to the surface of the bone tissue [139]. The acidification of this bone-osteoclast resorptive microenvironment and the deposit of proteases such as cathepsin K is achieved through the fusion of tissue-specific secretory lysosomes with the plasma membrane. It has been revealed that Atg proteins, which are part of the two conjugation systems including ATG5, ATG7, ATG4B, and LC3, play an important role in the fusion of these secretory lysosomes with the plasma membrane and subsequent formation of the ruffled border [140]. This process very likely does not represent a situation like the ones described above where autophagosomes fuse with the plasma membrane because secretory lysosomes are single membrane vesicles and their cargo is not cytoplasmic. It will be interesting in the future to determine whether *ATG* belonging to other Atg functional groups are also involved in this type of secretion.

A similar process involving the two Atg conjugation systems in organelle fusion has also recently been characterized in phagocytic cells and termed LC3-associated phagocytosis (LAP). In macrophages and other cell types uptaking apoptotic and necrotic cells, but also yeast or latex beads-conjugated LPS, LC3 is rapidly recruited to phagosomes in a manner that depends on ATG5, ATG7, BECLIN1, and hVPS34 [141–143]. Work in *C. elegans* on the same process has also implicate Atg18/WIPI [144]. While it is unclear whether BECLIN1 and hVPS34 are recruited as part of the autophagy-specific PtdIns3K complex I, what has been shown is that LAP does not require ULK1 and FIB200 revealing that the Atg1/ULK complex does not participate in this pathway [141, 142]. The translocation of LC3 onto phagosomes during LAP is not due to the fusion of autophagosomes with phagosomes as it has been observed for example during the killing of *Mycobacterium* [145], indicating a probable direct conjugation of LC3 to the limiting membrane of this latter organelle [142, 143]. Interestingly, LC3 association to phagosomes promotes their fusion with lysosomes leading to a rapid acidification and enhanced killing of the ingested organism [141–143].

Another documented case where it has been shown that cells have the capacity to use a portion of the Atg machinery (as part or not of another pathway) is the IFN*γ*-mediated antiviral response in macrophages [146]. In particular, it has been shown that the direct antiviral activity of IFN*γ* against murine norovirus, which involves an inhibition of the formation of the membranous cytoplasmic replication complexes of this virus, depends on the ATG5-ATG12 conjugate, ATG7 and ATG16L1, but not on the induction of autophagy, fusion between autophagosomes and lysosomes, and the degradative activity of lysosomes [146]. In addition, this response does not require the Atg8/LC3-processing protein ATG4B indicating that it uses just one of the two conjugation systems. It remains unclear how Atg5-Atg12 and Atg16L1 are carrying out their antiviral action, but interestingly Atg16L1 is detected on the norovirus replication complexes indicating that these proteins could affect the organization of the membranes harboring them and/or the replication machinery. Few questions still have to be answered in our way to understand the contribution of these Atg proteins in the direct IFN*γ*-mediated antiviral response. First, it must be determined whether ATG4B is substituted by one or more of its homologues (e.g., ATG4A, ATG4C, and ATG4D) before excluding the participation of the second conjugation system. Second, it will be interesting to analyze whether components of the other Atg functional groups are involved in this response.

## 6. Conclusion and Perspectives

The study of autophagy has attracted a lot of interest in the past years because of its multiple physiological and pathological implications. While major advances have been achieved in understanding the regulation, the mechanism, and the cellular roles of this versatile transport pathway, the new discoveries have unveiled new interesting aspects and their in-depth exploration will keep researchers busy for the next few decades. With the increasing number of laboratories starting to investigate autophagy in tissues, organisms, and diseases so far unexplored, it is easy to predict that the future will be full of surprises and autophagy will continue to astonish us for some time.
